# Insertions, deletions, and exchangeable couplings: a Dirichlet process over TKF92 domains and sites

**DOI:** 10.64898/2026.05.16.725674

**Published:** 2026-05-19

**Authors:** Annabel Large, Ian Holmes

**Affiliations:** Department of Bioengineering, University of California, Berkeley

## Abstract

The TKF92 model of molecular evolution—a linear birth-death process for indels, with finite-state continuous-time Markov chain substitutions—is exchangeable in residue identity at every site: the generative process treats amino acids symmetrically, conditional on a single substitution rate matrix. To introduce local heterogeneity, evolutionary models are often equipped with site-class mixtures, preserving this symmetry in the sense of de Finetti: conditional on the latent class, residues are still exchangeable. In a four-step theoretical ladder, we show how long-range structure such as couplings between distant sites can also be introduced exchangeably by using a Dirichlet process to partition sites into co-evolving classes. Our first step is a thorough analysis of TKF92 to establish sufficient statistics, limiting behavior, and inferential tools. We then lift the pairwise TKF92 hidden Markov model, in the limit of small time, to a *time-indexed gravestone-augmented pair stochastic context-free grammar*, and from there to its phylogenetic generalisation. This framing allows trajectories to be sampled exactly by Inside-Outside recursion. The third step places a Dirichlet process over the alive sites and asks co-keyed sites to evolve under a sparse Potts interaction — an exchangeably-partitioned hidden direct-coupling model whose marginal alignment likelihood is unchanged from plain TKF92. The fourth rung of the ladder develops inference machinery: a Gibbs–Metropolis sampler that alternates alignment resamples, key-partition resamples, and stochastic parameter updates. We close several gaps along the way — exact closed-form sufficient statistics for the linear birth–death–immigration component, the resolvable L’Hôpital limit at λ=μ, and a closed-form M-step for a recursive generalisation of TKF92 — and we report a 1,000-family Pfam fit with K=4 site classes whose Potts atoms carry ~0.54 nats of covariation per class-pair on top of a substantial single-site substitution model. Supplementary material, including full source code for inference, may be found at https://tkfdp.net/.

## 1 Exchangeability of indel models

The dominant frameworks of protein-sequence statistics are each limited in their own way. Substitution-only phylogenetic models (LG08, CAT, gamma-rates (24, 23, 37)) treat pre-aligned sites as independent given the tree; site–site epistasis is fitted away into an across-site rate-multiplier, not as a structural interaction. Direct-coupling analyses (plmDCA, mfDCA (25, 27, 9)) recover sharp contact predictions from joint amino-acid frequencies, but they treat the alignment as data and rely on multiple-sequence alignment tools whose own assumptions are broken by the same coevolution they want to detect. Indel birth-death models (TKF91/TKF92 (33, 34, 10) and the Poisson indel process (2)) sit somewhere between, with a cleanly separable framework for insertions, deletions, and point substitutions but no mechanism for coevolution between surviving sites.

**The full potential of exchangeability is going unused**. TKF92 is exchangeable in residue identity: the indel process treats amino acids symmetrically (insertions are drawn i.i.d. from a fixed distribution) and the substitution generator factorises over sites. A site-class mixture (23) breaks this exchangeability in a controlled way — two latent classes have two different stationary distributions, but the dynamics within a class are still identity-exchangeable. The Mixture-of-Domains generalisation of TKF92 (22) (MixDom) breaks it again by nesting geometric fragments inside a top-level domain HMM. Both elaborations preserve a key feature: *conditional on the latent class / domain / fragment, residues are still exchangeable*.

This is our starting point for indel-aware coevolution. Each newly inserted residue is assigned a random “key”: shared keys indicate coupling partners. The key assignment is exchangeable in the sense of de Finetti: the key labels are latent, and the distribution over them is invariant to permutation of the sites. The same is true of the site-class labels that govern the single-site substitution dynamics. Once those labels are sampled, the substitution rates are determined symbolically, and indels are independent of all of them.

The contributions are organised as a four-rung ladder:

1. **Rung** 1 (Section 2.1). *Exchangeably covariant TKF92.* Closed-form BDI sufficient statistics and closed-form Baum–Welch M-steps for TKF91, TKF92, and the singly-recursive MixDom; the L’Hôpital limit at λ=μ closes the numerical singularity.
2. **Rung 2** (Section 2.2). *Posterior over evolutionary trajectories.* Gravestone-augmented TKF92 in its infinitesimal-time limit is a continuous-time-indexed pair SCFG; its Volterra fixed-point is a bivariate Riccati on alive and gravestone counts, closed-form inside-outside, lifting to a *phylogenetic* SCFG via Felsenstein-style upwards–downwards.
3. **Rung 3** (Section 2.3). *Exchangeable partitioning via Dirichlet processes.* A Chinese-restaurant draw partitions alive sites into co-keyed cliques; clique-mates share a Potts coupling on top of a per-class GTR generator, governed by three independent DPs (keys, classes, Potts atoms). The marginal alignment likelihood is unchanged from rung 1.
4. **Rung 4** (Section 2.4). *Inference by variational EM and MCMC.* Gibbs-within-stochastic-VI: inner E-step for the clique CTMC; Jain–Neal sweeps over the three partition latents; recursive-traceback resample of the gravestone-augmented branch history; closed-form M-step into profile, Potts atom, and rate multiplier.

## 2 Methods at a glance

### 2.1 Rung 1: closed-form TKF foundations

#### The BDI process and its sufficient statistics.

**Figure 1:**
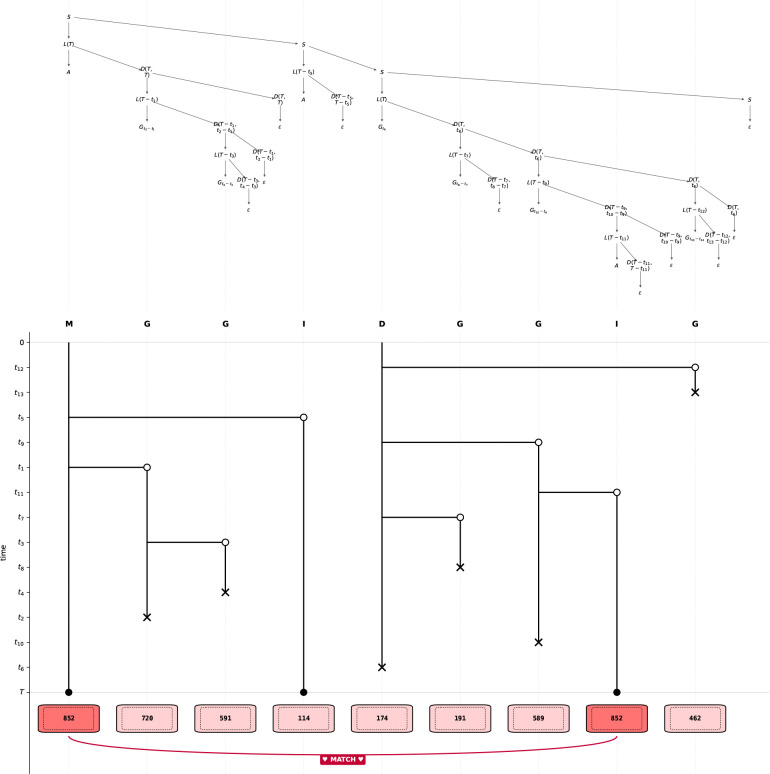
TKF91 indel trajectory as a Galton–Watson branching process and its gravestone-augmented SCFG parse tree. *Top:* the gravestone-augmented SCFG parse tree, with each non-terminal annotated by its symbolic time arguments (t,T) in terms of the event indices $t_{1},\ldots ,t_{13}$ that label the middle panel’s vertical axis. Each L(·) leaf-terminal (A for surviving lineages, $G_{\tau }$ for transient ones) sits directly above its corresponding trajectory column; the faint dotted vertical guides carry the eye from each parse-tree terminal down through the trajectory column and into its lottery ticket. Epsilon leaves (ε) terminate D-recursion and S-recursion and are placed mid-way between their column-anchored neighbours. The event labels $t_{k}$ are assigned in fixed pre-order traversal of the tree, so the parse-tree subscripts are deterministic; the random topological sort over event times permutes only the vertical axis ordering of the middle panel. *Middle:* one realisation of the continuous-time TKF91 process on a branch of length T. Columns are residue lineages, time runs downward from 0 to T. Open circles (◦) mark birth events; filled circles (•) mark survival to T; crosses (×) mark death events. The ancestral marker M (col. 1) survives to T and spawns a transient insert G (col. 2; lifetime $t_{1}\to t_{2}$), which itself spawns a deeper transient G (col. 3; $t_{3}\to t_{4}$). The insertion I (col. 4) is born at $t_{5}$ and survives. The ancestral site D (col. 5) dies at $t_{6}$ after spawning three transient inserts (cols. 6, 7, 9; lifetimes $t_{7}\to t_{8}$, $t_{9}\to t_{10}$, $t_{12}\to t_{13}$); the second of these transients itself spawns the surviving insertion I (col. 8) at $t_{11}$. The horizontal arrows depict the SCFG branching production D(t,T)→L(t−u)·D(t,T) firing at each child link’s birth; the immortal link (dashed) anchors the leftmost lineage. *Bottom:* a row of red lottery tickets cartooning the TKF-DP Potts-coupling layer: each surviving column draws a random integer “key” (here a three-digit number) from the Chinese restaurant process partition. Most keys are unique, but two distant alive columns happen to share a key (the MATCH), which is precisely the event in which two sites end up at the same CRP table and acquire a Potts coupling under the K=4 model of Section 2.3.

TKF91 places one linear birth–death–immigration process on the sequence length, with insertion rate λ, deletion rate μ, and immigration coincident with insertion. The endpoint-conditioned expected sufficient statistics — the birth count B, the death count D, and the time-integrated alive count S — have a closed form via the Fisher score identity applied to the Kendall–Karlin–McGregor transition density (20). The sufficient statistics implicitly account for the gravestone lineages and other transient trajectory segments unobserved at the endpoints (Figure 1). Writing p(j|i;t)=E[1{N(t)=j}|N(0)=i] for the BDI transition density, differentiating logp with respect to λ and μ gives two score identities involving E[B],E[D], and E[S]; the Karlin–McGregor count-balance j−i=E[B]−E[D] closes the system. Solving:

(1) $$\begin{array}{l} E[S|i,j,t]\;=\frac{(j\;-\;i)\;+\;\mu \;\partial_{\mu }\;log\;p(j\;|\;i;\;t)\;-\;\lambda \;\partial_{\lambda }\;log\;p(j\;|\;i;\;t)\;-\;\lambda t}{\lambda -\mu },\\ E[B|i,j,t]\;=\lambda \partial_{\lambda }\log p\left(j|i;t\right)+\lambda E\left[S|i,j,t\right]+\lambda t,\\ E[D|i,j,t]\;=\mu \partial_{\mu }\log p\left(j|i;t\right)+\mu E\left[S|i,j,t\right]. \end{array}$$

**Figure 2:**
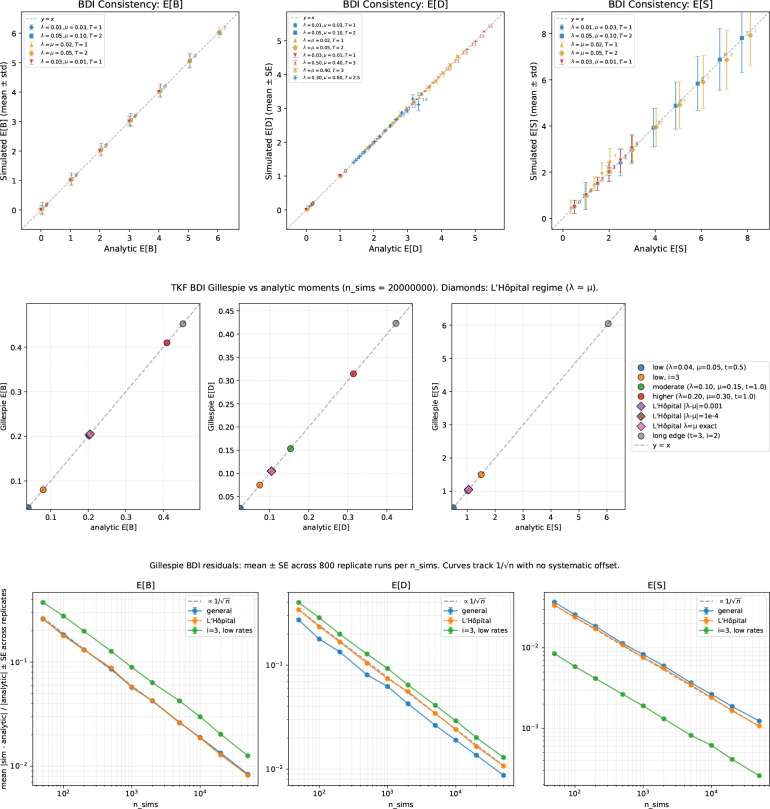
BDI / TKF sufficient-statistic validation. Top row, panels (a)–(c): closed-form score-identity expectations (analytic) plotted against Gillespie sample means (simulation, 10^7^ trajectories per regime at low rates; 10^6^ trajectories at higher rates) for births B (left), deaths D (middle), and time-integrated alive count S (right). Each point is one ($\lambda ,\mu ,t,i_{0}$) regime; the identity-line scatter is below plotting precision across κ<1, κ=1 (L’Hôpital diamonds), and κ>1. The five low-rate regimes (λ,μ~0.01−0.10,t~1) sweep E[D] over ~0–1 (most trajectories see at most one death); three additional higher-rate regimes (λ,μ~0.3−0.6,t~2.5−3) extend the D panel through E[D]≈5, exercising the score identity across an order of magnitude in event count without bias. Numerals beside each point are the descendant count *j* that bin represents; residual scatter at the largest j reflects per-bin MC noise that shrinks as $1/\sqrt{n_{\text{bin}}}$. Error bars are standard errors of the mean (SE $=\sigma_{\text{sim}}/\sqrt{n_{\text{bin}}}$). Panel (d): TKF91/TKF92 BDI moments via Gillespie (2 × 10^7^ sims per regime, 5 regimes) against the closed-form expressions; tight identity-line. Panel (e): relative Gillespie residual scaling as $1/\sqrt{n_{\text{sim}}}$ over 800 replicate seeds per $n_{\text{sim}}$, three representative regimes (general, L’Hôpital, low-rate $i_{0}=3$); no detectable offset.

The E[S] formula has a 0*/*0 singularity at λ=μ: the (λ−μ) denominator vanishes, and the numerator vanishes too as an automatic consequence of the same count-balance identity that closed the system. Earlier work (7, 12) patched the singularity with an ε-clamp; it is in fact a resolvable L’Hôpital limit recovered exactly by differentiating numerator and denominator a second time in λ−μ, matching Gillespie simulations to Monte-Carlo precision at 10^7^ trajectories per regime (Figure 2). The same identity applied to the TKF92 fragment-level process supplies the closed-form sufficient statistics for the indel side of TKF92 and of MixDom.

#### Closed-form Baum–Welch for TKF, including recursive variants.

With sufficient statistics in hand, the M-step for TKF91 and TKF92 is closed form: an exact quadratic equation in (λ,μ,r) from the per-cherry counts, plus a standard GTR update on the substitution side from bridge expectations (14). Recursive variants—such as the previously-described singly-recursive MixDom model of proteins (22) and fully-recursive TKF Structure Tree models of RNA structure (11), or other models of RNA, protein, and genome evolution described in the appendix—present a separate subtlety: their potentially-empty nested substructures require null state elimination by Schur complement. Naively, the M-step then needs second derivatives through the eliminated null blocks. We show how to restore eliminated counts exactly by a systematic backward elimination chain: the Forward–Backward (Inside–Outside) counts on the collapsed pair HMM (or pair SCFG) are pushed backward to per-null counts on the exploded model, and a single sum-by-parameter-group recovers unbiased BDI sufficient statistics for every parameter. The algebra is verified against autodiff at 10^−6^ relative tolerance for MixDom; the practical consequence is that rate-space Baum–Welch is exact at every level (top-level domain, per-domain fragment, per-fragment class, weights).

#### Single-site substitution.

The substitution generator is the F81 instance of GTR, $Q_{xy}^{\left(c\right)}=\eta_{s}S_{xy}\pi^{\left(c\right)}(y)$, with a shared exchangeability matrix $S\in R^{20\times 20}$, a per-class stationary distribution $\pi^{\left(c\right)}\in \Delta$^19^, and a per-site gamma rate multiplier $\eta_{s}$. The F81 form is one of two natural reversible instances; we adopt it (over the symmetric Metropolis form) because it admits strict Dirichlet–multinomial conjugacy on $\pi^{\left(c\right)}$ via a secret-destination augmentation of the embedded random walk (Section 2.4). Class assignments are drawn from a Dirichlet process with concentration $\alpha_{c}$; the limit reproduces the CAT profile mixture (23) and, augmented with the $\eta_{s}$ multiplier, the Yang gamma-rates model (37).

### 2.2 Rung 2: the time-indexed gravestone-augmented pair SCFG

#### From pair HMM to time-indexed pair SCFG.

TKF91, viewed as a Markov process on sequence length and observed through a pair HMM at finite time t, has a continuous-time embedding that is not itself a pair HMM but a stochastic context-free grammar with productions indexed by time. Let L(t) denote one link of age t; under TKF91 dynamics, L(t) has lifetime τ~Exp(μ) and spawns child links at rate λ during its lifetime. The branching structure is a continuous-time Galton–Watson process expressible as

$$\begin{array}{llll} \;L(t)\to A\cdot D(t,t) & \; & & \text{weight}\;e^{-\mu t},\\ \;L(t)\to G_{\tau }\cdot D(t,\tau ) & \; & & \text{density}\;\mu e^{-\mu \tau }d\tau ,\;\tau \in \left(0,t\right),\\ D(t,T)\to L(t-u)\cdot D(t,T) & \; & & \text{density}\;\lambda du,\;u\in \left(0,T\right),\\ D(t,T)\to \varepsilon & \; & & \text{weight}\;e^{-\lambda T}. \end{array}$$

The terminals A and $G_{\tau }$ are an *alive* residue and a *gravestone* residue that died on the branch at relative time τ; D(t,T) generates a list of child links born during a window of size T inside an observation interval of size t. The TKF92 expansion is mechanical: replace each single-residue terminal with a geometric fragment of shared death time, leaving the fragment-level branching grammar unchanged. The pair version adds an ancestor strand on the left of each $A/G_{\tau }$ terminal; insertions have no ancestor counterpart. The grammar parse tree is the continuous-time embedding of the TKF trajectory (Figure 1): each L(t) non-terminal expansion corresponds to one branch of the Galton–Watson process, and the $A/G_{\tau }$ terminals are the alive and gravestone residues whose lifetimes the parse tree records.

#### Why the augmentation matters: transient lineages.

Under any model with site–site coupling, an alignment-invisible fragment — born and deleted on the same branch — still changes the substitution dynamics of every site it shares a coupling partner with during its lifetime. The naive scheme of sampling only the alignment-visible indel events misses these transient lineages. The augmentation places them explicitly in the grammar’s parse trees, as $G_{\tau }$ residues with finite lifetimes, so that the substitution likelihood can sum over them. The instantaneous alive count $N_{a}(t)$ is bounded pathwise by the visible-at-T count plus the gravestone count, so the indel CTMC bridge has a finite state space and admits a closed-form recursive sampler.

#### The bivariate Riccati.

Let $f_{t}(z,w)=E\left[z^{N_{a}(t)}w^{N_{g}(t)}\right]$ be the joint generating function for alive count $N_{a}$ and gravestone count $N_{g}$. The Volterra fixed point

(2) $$f_{t}\left(z,w\right)=z\;e^{-\mu t}\exp\left(\lambda \int_{0}^{t}\;\left(f_{s}\left(z,w\right)-1\right)ds\right)+w\int_{0}^{t}\mu e^{-\mu \tau }\exp\left(\lambda \int_{t-\tau }^{t}\;\left(f_{s}\left(z,w\right)-1\right)ds\right)d\tau ,$$

is equivalent to the forward equation $\partial_{t}f_{t}=\left[\lambda z^{2}-(\lambda +\mu )z+\mu w\right]\partial_{z}f_{t}$ with $f_{0}=z$, a Riccati in z whose flow closes algebraically. The Volterra fixed-point above is the Laplace transform of the Inside algorithm of stochastic context-free grammars (21, 30, 15): the SCFG production weights of the time-indexed grammar enter as their integrated kernels (survival $e^{-\mu t}$ and rate-density $\mu e^{-\mu \tau }d\tau$), and inside-outside in the (z,w) generating-function domain is the fixed-point iteration of the Volterra equation. Defining $\Delta (w)=\sqrt{\left(\lambda -\mu {)}^{2}+4\lambda \mu (1-w\right)}$ and $z_{\pm }(w)=((\lambda +\mu )\pm \Delta (w))/(2\lambda )$, the flow $\left(z(t)-z_{+}\right)/\left(z(t)-z_{-}\right)=\left(z(0)-z_{+}\right)/\left(z(0)-z_{-}\right)\cdot e^{-\Delta (w)t}$ inverts to give pointwise transition probabilities for ($N_{a},N_{g}$) at any branch length. Setting w=1 recovers the standard linear birth–death PGF for $N_{a}$ alone.

#### Phylogenetic lift.

The per-branch closed-form transition kernel from the Riccati composes across branches: the marginal at each internal node, conditional on the rest of the tree, is a closed-form pointwise distribution computable in O(1) given the per-branch kernel evaluations. Felsenstein-style upwards–downwards then gives a tree-level closed-form sampler for the gravestone-augmented history — a *phylogenetic* SCFG of the same time-indexed form. Earlier work on pair SCFGs (32, 21, 15, 30) operates at fixed time; the present construction is the infinitesimal limit, and it lifts to trees without modification.

### 2.3 Rung 3: Dirichlet-process partitioning and Potts couplings

#### The TKF-DP model.

Fix TKF92 indel parameters (λ,μ,r), a key-class concentration $\alpha_{z}>0$, a site-class concentration $\alpha_{c}>0$, a Potts-atom concentration $\alpha_{H}>0$, and base measures $G_{0}$ on $\pi^{\left(c\right)}$ (Dirichlet) and $G_{0}^{H}$ on entries of each Potts atom $H_{h}\in R^{20\times 20}$ (Gaussian).

#### Definition 1 (TKF-DP).

Sample stick-breaking weights for three independent Dirichlet processes (one over key classes, one over site classes, one over Potts atoms). Run TKF92 dynamics with parameters (λ,μ,r). At each fragment-birth event, draw ($z_{s},c_{s},\eta_{s}$) from $\left(Cat\left(\pi^{z}\right),Cat\left(\pi^{c}\right),Gamma\left(a_{\eta },b_{\eta }\right)\right)$ for each new residue; the labels are fixed for the residue’s lifetime. At each first co-occurrence of an unordered class-pair $\left\{c,c^{\prime }\right\}$, assign it a Potts-atom index $h_{cc^{\prime }}∼Cat\left(\pi^{H}\right)$, materialising an atom $H_{h_{cc^{\prime }}}$ from $G_{0}^{H}$ if new. The amino-acid trajectory on each key class C is the single-site reversible CTMC with rate

$$Q^{s}\left(x\to x^{\prime };x_{C\backslash s}\right)=\eta_{s}S_{xx^{\prime }}\pi^{\left(c_{s}\right)}\left(x^{\prime }\right)exp\left(-\frac{1}{2}\sum_{s^{\prime }\in C\backslash s}\;\left[H_{c_{s}c_{s^{\prime }}}\left(x^{\prime },x_{s^{\prime }}\right)-H_{c_{s}c_{s^{\prime }}}\left(x,x_{s^{\prime }}\right)\right]\right),$$

which is reversible with respect to the joint Potts stationary

$$\pi_{C}\left(x_{C}\right)\propto \prod_{s\in C}\pi^{\left(c_{s}\right)}\left(x_{s}\right)\cdot exp\left(-\sum_{\left\{s,s^{\prime }\right\}\subset C}\;H_{c_{s}c_{s^{\prime }}}\left(x_{s},x_{s^{\prime }}\right)\right).$$

#### Alignment–substitution factorisation is preserved.

The key DP draws are i.i.d. across sites at birth, independent of the alignment path and of the substitution history. The joint distribution therefore factorises as

(3) $$P\left(\text{align},𝒫,\left\{z_{s}\right\},\;\text{subst}\right)=P_{TKF}(\text{align})\cdot P\left(𝒫|L_{A}\right)\cdot \prod_{s}P\left(z_{s}|𝒫\right)\cdot P\left(\text{subst}|\text{align,}\left\{z_{s}\right\}\right),$$

where $L_{A}:=|Alive(align)|$ is the number of alive sites the alignment exposes (for the pairwise object of Equation (5): the Match cells); the partition prior depends only on this count, not on alignment geometry. In particular: the marginal alignment likelihood is exactly TKF92’s at every $\alpha_{z}$; the $\alpha_{z}=\infty$ all-singletons limit recovers TKF92 + per-site GTR pointwise. The coupling enters only through the substitution factor.

#### Partition statistics and identifiability.

Marginalising ${𝒫}^{z}$, the partition on any finite set of L sites is the Ewens partition with parameter $\alpha_{z}$, equivalent to a Chinese restaurant process draw. The expected total number of classes among L sites is $O\left(\alpha_{z}\;log\;L\right)$, with k-cliques suppressed by $1/\alpha_{z}^{k-1}$ relative to the all-singleton baseline; the prior is naturally sparse. The three DPs play distinct roles. $\alpha_{c}$ controls the number of materialised site classes; $\alpha_{z}$ controls the per-MSA partition into Potts cliques; and $\alpha_{H}$ controls identifiability of the Potts side. Without an atom DP, the free parameter count is $O\left(K_{c}^{2}\cdot A^{2}\right)$ for $K_{c}$ site classes and A-state alphabet; with it, the count is $O\left(K_{H}\cdot A^{2}+K_{c}^{2}\right)$ class-pair indices, where $K_{H}$ is data-driven and typically much smaller than $K_{c}^{2}$. Distinct class-pairs $\left\{c,c^{\prime }\right\},\left\{d,d^{\prime }\right\}$ may share an atom index $h_{cc^{\prime }}=h_{dd^{\prime }};K_{H}$ counts distinct atoms, not distinct class-pairs, and the atom DP’s reinforcement is what drives this sharing. We use $K_{H}\leq K_{c}\left(K_{c}+1\right)/2$ truncated stick-breaking by default; at $K_{H}=K_{c}\left(K_{c}+1\right)/2$ the truncation is tight (every class-pair could in principle have its own atom). Dirichlet processes have previously been explored to partition MSA columns into site classes and rows into functional classes (17, 16, 4, 28, 31). The CRP draws on key classes $\left\{z_{s}\right\}$ are the “lottery tickets” of Figure 1: each residue draws a key at birth, residues sharing a key are coupled, and the Ewens prior on the resulting partition is what selects which columns enter a Potts clique.

#### Connection to direct-coupling analysis.

Direct-coupling analyses (25, 27, 9) fit a Potts model on the joint amino-acid frequencies of aligned columns of a single protein family. The TKF-DP is the indel-aware, phylogenetically-explicit, exchangeably-clustered generalisation: it conditions on a generative model of how aligned columns came to exist (TKF92 indels through time on a tree), it accumulates coevolutionary evidence across a corpus through a shared Potts-atom DP rather than per-family, and the columns it couples are drawn from an Ewens-distributed cluster prior rather than fitted as $O\left(L^{2}\right)$ explicit dyadic edges. The Wilburn–Eddy hidden Potts model (36), designed for remote homology search, is the closest single antecedent: it pairs a profile-HMM-style indel architecture with a Potts model over residue identities and makes the same architectural commitment that coevolution and indels must be scored jointly rather than as post-hoc rescoring.

### 2.4 Rung 4: inference

The inference loop combines four ingredients in a stochastic variational EM scheme. We give each in one paragraph; full derivations are in the supplement.

#### Inner variational E-step.

For each branch and each key DP class, the joint substitution dynamics on ${𝒜}^{\left|C\right|}$ has no usable eigendecomposition for |C|>1, and direct matrix exponentiation is infeasible for |C|>2. We approximate by the path-measure mean-field of Cohn et al. (6), restricted to a constant per-site rate matrix ${\overset{ˆ}{Q}}_{b}^{s}$ on each composition-constant segment. The geometric-mean fixed point $log\;{\overset{ˆ}{Q}}_{b}^{s,*}\left(x\to x^{\prime }\right)=log\;\eta_{s}+log\;S_{xx^{\prime }}+log\;\pi^{\left(c_{s}\right)}\left(x^{\prime }\right)-\frac{1}{2}\sum_{s^{\prime }\in C\backslash s}E_{{\overset{‾}{q}}_{b}^{s^{\prime }}}\left[H_{c_{s}c_{s^{\prime }}}\left(x^{\prime },x_{s^{\prime }}\right)-H_{c_{s}c_{s^{\prime }}}\left(x,x_{s^{\prime }}\right)\right]$ admits a closed-form ELBO via pairwise bridge expectations $\left(Br(\alpha ,\beta ,t)=\left(e^{\alpha t}-e^{\beta t}\right)/(\alpha -\beta )\right.$ for $\alpha \neq \beta ,=te^{\alpha t}$ otherwise). The constant-rate scheme is the N=1 end of an adaptive refinement that converges to Cohn’s full inhomogeneous family as N→∞ sub-segments; the gravestone augmentation of rung 2 supplies a forced segmentation at every composition-change event.

#### MCMC over the discrete and augmentation latents.

Three combinatorial latents alternate. Augmented branch histories can be resampled by recursive midpoint traceback on the time-indexed pair SCFG of rung 2, with closed-form midpoint marginals from the bivariate Riccati and Felsenstein-style upwards–downwards across the tree. Key DP labels $z_{s}$ flip by Chinese-restaurant Gibbs with class-level ELBO ratios from the previous variational step; site classes $c_{s}$ flip by analogous CRP-Gibbs with single-site likelihoods; Potts-atom indices $h_{cc^{\prime }}$ resample by truncated stick-breaking sweeps that share atoms across class-pairs. Jain–Neal split–merge proposals (18) on all three partitions provide global mixing. Pairwise alignment to the Infinite Pair HMM (1) uses a per-MSA block-resample on site partitions, with a setup-phase $O\left(L^{4}\right)$ precompute of the two-anchor partial-Forward tensor and per-sweep cost O(L) (amortised: each sweep does O(1) inter-edge-anchor segment resamples, each an $O\left(L_{\text{seg}}\right)$ stochastic traceback through the cached partial-Forward).

#### Closed-form base-measure integrals.

The new-class predictives in the three CRPs require integrals against the base measures. Three structural conjugacies make them closed form: a *secret-destination augmentation* on $\pi^{\left(c\right)}$ (the F81 rate factors as an S-driven Poisson proposal clock with a $\pi^{\left(c\right)}$-driven Bernoulli filter, and silent-failure votes—including transitions back to source as an unobserved destination—complete the multinomial structure) gives Dirichlet–multinomial conjugacy on the per-class profile; a Gamma–Poisson conjugacy on $\eta_{s}$ gives closed-form Negative-Binomial site marginals; and a Gaussian-Laplace approximation around the path-DCA MAP gives closed-form Gaussian posteriors on each Potts atom, with a small multi-seed Laplace mixture for atoms with multimodal basins.

#### Pairwise posteriors for decision-theoretic multiple alignment.

For downstream consensus-MSA assembly via sequence annealing (3), we expose a per-Match-cell posterior $Q_{ij}^{\prime }$ derived from the joint-marginal alignment-and-partition target of Equation (5): an alignment *A* together with a size-{1,2} partition E of its Match cells contributes a product of per-block factors — $P_{\text{singlet}}\left(x_{i},y_{j};t\right)$ at unpartnered Match cells and $P_{\text{doublet}}\left(\left(x_{i},x_{k}\right),\left(y_{j},y_{l}\right);t\right)$ at coupled doublet endpoints — with class assignments and rate multipliers integrated analytically rather than re-marginalized at every column. The supplement details the per-block primitives, the augmented-Pair-HMM bounded-edge family ($O\left(L^{2}A^{2}\right)$ for 1-edge, $O(L^{2}A^{4})$ for 2-edge), and the infinite-Pair-HMM MCMC sampler that targets the unbounded-edge limit.

#### Counts-tensor training corpus.

Per-family int8 (cherry × column × AA-pair × branch-bin) count tensors decouple inference from MSA parsing, letting one SVI outer iteration scale to 10^3^–10^4^ Pfam families.

## 3 Results

### 3.1 Closed-form sufficient statistics match Gillespie simulation

The BDI sufficient-statistic formulae of Equation (1) agree with event-driven Gillespie simulation to within Monte-Carlo precision at $n_{sim}=10^{7}$ trajectories per ($\lambda ,\mu ,t,i_{0}$) regime. Figure 2 panels (a)–(c) show closed-form score-identity estimates against Gillespie sample means for the birth count B, death count D, and time-integrated alive count S across a grid spanning κ=λ/μ∈{<1,1,>1}. The L’Hôpital limit at λ=μ has no *ε*-clamp degeneracy at the diagonal and matches Gillespie to Monte-Carlo precision. Lifting to the TKF91/TKF92 process and to the full forward–backward E-step, panel (d) shows the relative Gillespie residual scaling as the canonical $1/\sqrt{n}$ across three representative regimes, with 800 replicate seeds per $n_{\text{sim}}$ and no detectable bias offset — confirming both unbiasedness of the score-identity expressions and statistical correctness of the Gillespie simulator.

### 3.2 Pfam training signals: covariation and rate heterogeneity

#### Covariation signal.

**Figure 3:**
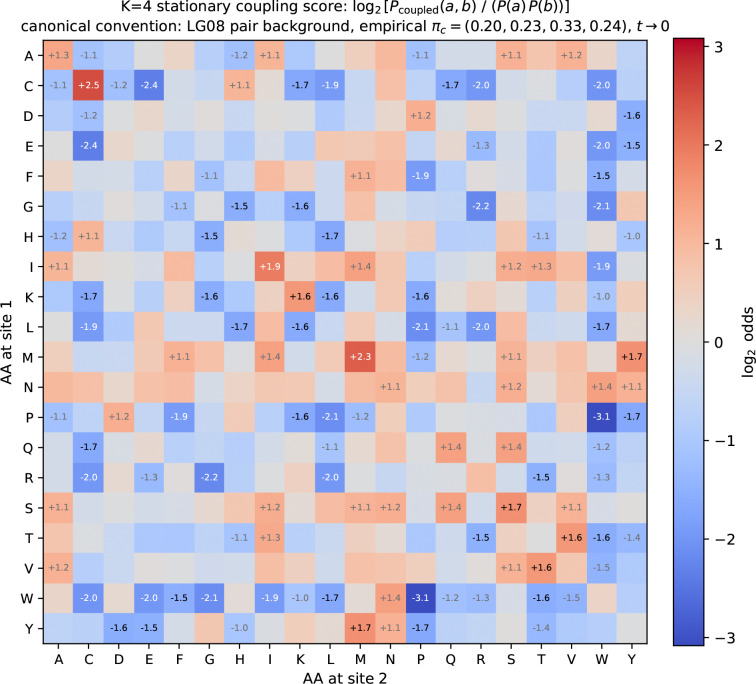
Stationary coupling log-odds at a coupled site-pair in the K=4 TKF-DP model. Cell (a,b) shows ${log}_{2}\left[\pi_{\text{joint}}(a,b)/\left(\pi_{\text{singlet}}(a)\pi_{\text{singlet}}(b)\right)\right]$ in the t→0 limit, under the canonical convention (LG08 pair-stationary background + empirical class prior $\pi_{c}=(0.20,0.23,0.33,0.24)$ from SVI training-set column assignments). *Red* cells are enriched at coupled sites relative to the singlet product (positive log-odds); *blue* cells are depleted. The strongest positive cells are C–C (+2.51 bits; the disulfide-bridge signature: cysteine pairs co-occur far more often at structurally coupled positions than background frequencies predict), M–M (+2.32), and I–I (+1.93); the strongest off-diagonal positive is M–Y (+1.66), consistent with the methionine–aromatic (sulfur–π) stabilising interaction documented in folded structures (35). The strongest negative cells include several pairs whose Chou–Fasman secondary-structure propensities are antagonistic — P–W (−3.08), L–P (−2.12), K–P (−1.63) all pair the helix/strand breaker proline with strong helix or strand formers (5) — though the pattern is not uniformly explained by secondary-structure preferences alone: top empirical negatives also include C–E (−2.36), G–R (−2.21), and a Trp column with broad aversion that the low Trp background frequency (π(W)≈0.011 in LG08) likely inflates. Mean per-class-pair MI under this convention is ~0.54 nats / 0.78 bits (atom-averaged).

A $K_{c}=4,K_{H}=10$ TKF-DP fit to 1,000 Pfam families (26) (indel rates pre-trained by Maraschino cherry-distillation (29) and fixed; 500 EM warmup iterations on the Dirichlet–multinomial class assignment, then 55 stochastic VI outer iterations with the full coupled loop; MSAs as the only supervision, no PDB) recovers a covariation signal visible directly in the stationary class-pair log-odds heatmap (Figure 3): a +2.51-bit Cys–Cys disulfide signature on the diagonal, a +1.66-bit off-diagonal Met–Tyr attraction consistent with the methionine-aromatic motif, and a band of Chou–Fasman-consistent proline aversions on the negative side.

#### Indel and compositional heterogeneity.

Separately, a three-domain single-fragment MixDom model (d3f1) fit to the same corpus via SVI–BW with the exact closed-form chain-restoration M-step (Section 2.1; a Maraschino warm-start was tried but did not improve held-out LL) separates its three domains cleanly: one high-indel (λ≈0.11, μ≈0.17, weight 0.32, mean fragment length 2.8) with the classic disordered / loop signature (enriched in S, P; depleted in W, C, M, H, Y; mean Kyte–Doolittle −0.82), and two low-indel domains (~20× smaller rates, mean KD +0.07 and −0.22; enriched in L, V, I — the buried-core hydrophobic residues absent from the high-indel domain’s enriched set). Indel rate tracks hydrophilicity in the direction biological intuition predicts — mobile loops drift in length faster than buried cores — though most of the contrast is carried by the high-indel domain.

### 3.3 Pairwise alignment accuracy on structural benchmarks

#### Evaluation by expected pair-confusion.

We score pairwise alignment accuracy through the soft confusion matrix on the residue-pair cell grid: for each pair of sequences in a reference MSA guided by experimentally-determined structures, we read off the gold match set $T=\left\{\left(r_{i},r_{j}\right)\right\}$ from the curated alignment and compute, against the model’s $L_{i}\times L_{j}$ match-posterior matrix P, the sufficient statistics

(4) $$e_{TP}=\sum_{\left(r_{i},r_{j}\right)\in T}P\left[r_{i},r_{j}\right],\;\text{total}\;\text{mass}=\sum_{r_{i},r_{j}}P\left[r_{i},r_{j}\right]=e_{TP}+e_{FP},\;|T|=e_{TP}+e_{FN}.$$

These four numbers are additive across pairs and across families and suffice to compute soft precision, recall, and expected pair F1. The measurement is exact given the posterior; no Viterbi decoding is required, and no MSA reconstruction is performed.

#### BAliBase results.

Table 1 summarises pairwise expected-$F_{1}$ results on the 22-family BAliBase BAli3pdbm L<150 subset (187 sequence pairs) for the canonical pipeline: TKF92 baseline (single-component, corpus-fitted indel rates), TKF92-K=20 indel mixture, CherryML-C=20 per-site substitution-class mixture (single TKF92, marginalised over C=20 emission components), MixDom-d3f1, external aligners MAFFT (19) and MUSCLE (8), and the infinite-Pair-HMM (TKF-DP K=4 Potts-coupled) MCMC sampler. The MixDom-d3f1 model (D=3 domains, F=1 fragment per domain) is amongst the smallest MixDom models that meaningfully improve over plain TKF92 on this benchmark; adding fragments or classes beyond this scale gives diminishing returns. The infinite-PHMM-K=4 sampler uses the released checkpoint trained on ~1,000 Pfam families with EM warmup (Section 3.2); its column entries are the running-mean cell posterior $Q^{\prime }$ from the MCMC sampler (the 22-family L<150 subset is the practical eligibility ceiling for the $O\left(L^{4}\right)$ partial-Forward precompute on an 11 GiB GPU; see Section 3.2 for the calibration). The TKF-DP coevolutionary coupling is weak in the pairwise case, and appears to sharpen alignment accuracy only slightly, with gains lost by the sequence-annealing algorithm for MSA assembly (which does not, at present, share coupling signal across MSA columns, unlike the composite-likelihood method discussed in Section 3.4 and Figure 5).

### 3.4 The infinite Pair HMM as the natural fixed point

The methods of Table 1 together span the |E|=0 truncation (the soft-DP rows, which factor over Match cells and ignore Potts coupling between sites) and the |E|→∞ limit (the infinite-Pair-HMM sampler row, which couples sites via the size-{1,2} Ewens prior at the trained $\alpha_{z}$); they are progressively faithful approximations to a single underlying object: the alignment-and-partition posterior

(5) $$\pi \left(A,{𝒫}_{M}|X,Y\right)\propto \pi_{TKF92}(A|X,Y)\cdot \pi_{CRP}\left({𝒫}_{M}\left|\alpha_{z},|Match(A)|\right)\cdot \prod_{b\in {𝒫}_{M}}\;P_{block}(b|t,H),\right.$$

where ${𝒫}_{M}$ partitions the Match cells into singletons (block of size 1, contributing the standard per-site CTMC factor) and pairs (block of size 2, contributing the joint Potts CTMC factor), with the size-{1,2} truncation of the Ewens prior. The factorisation makes the alignment, partition, and substitution factors independent given each other; the block-resample MCMC kernel of Section 2.4 samples from Equation (5) exactly. Equation (5) is itself the principled single-branch marginal of the infinite gravestone-augmented phylogenetic SCFG (rung 2 of the ladder): in the full multi-branch object, the same CRP rule governs edge spawn at every site of every fragment — alive or gravestone — at every internal node, with the substitution likelihood depending on the full augmented history rather than only the alignment-visible fragments (Figure 1).

#### Worked example: divergent disulfide-rich pair from a held-out family (Figures 4 and 5).

**Figure 4:**
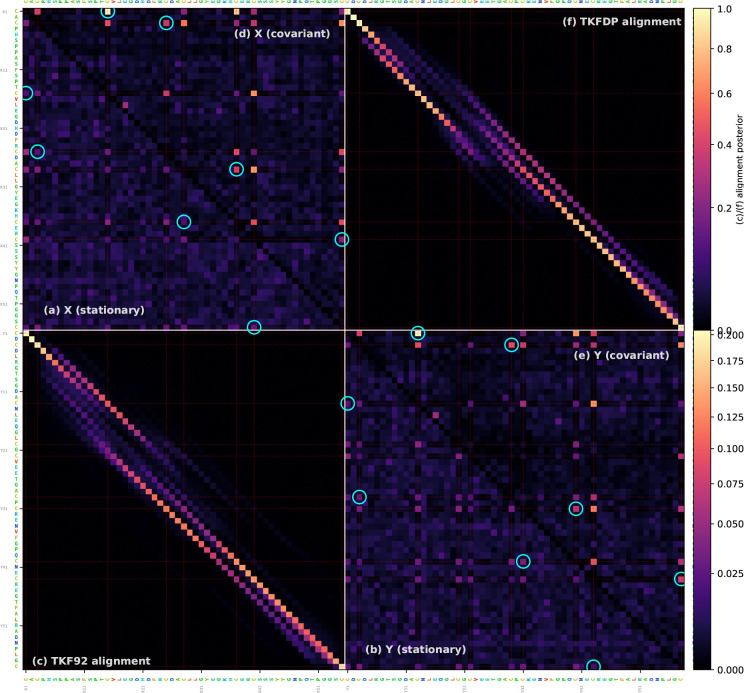
Coevolutionary signal boosts Cys–Cys disulfide bond identification on a sequence pair from a held-out test family. Sequences X, Y are two EGF-like repeats of human **LAMA1** (laminin subunit α−1, UniProt P25391; Pfam Laminin EGF-like family PF00053, held out from K=4 training). X is LAMA1 residues 1452–1506 ($L_{x}=55$, 8 Cys forming 4 UniProtannotated disulfides (circled) at seq-pos (1, 15), (3, 25), (28, 37), (40, 55)); Y is the LAMA1 repeat two positions earlier, residues 1090–1147 ($L_{y}=58$, 10 Cys: the same 4 disulfides at seq-pos (1, 13), (3, 29), (31, 40), (43, 58) plus 2 *free* Cys at LAMA1 positions 1109 and 1111 not in any annotated disulfide). *Panels (c, f):* TKF92 baseline alignment posterior (lower-left, lower-right) vs the TKF-DP alignment posterior after coupled-edge resampling (upper-right); the diagonal is sharpened by the coupling boost on conserved disulfide cells. *Panels (a, b):* single-sequence X/Y edge posteriors (no homolog evidence; Cys–Cys cells receive only the per-pair model boost and the matching-constraint prior). *Panels (d, e):* joint sampler edge posteriors at the same Cys positions, with the homolog providing positional discrimination across the diverged alignment. The joint sampler concentrates posterior mass at Cys–Cys cells at ~17× the background rate on the X axis (0.074 vs 0.004) and ~12× on Y (0.047 vs 0.004); the single-sequence baselines give only ~5× each. Sampler: joint pair replica-exchange ladder $\alpha_{z}\in \left\{100,250,700,2000,10^{4}\right\}$, 8000 sweeps + 1500 burn-in; single-sequence ladder $\alpha_{z}\in \left\{100,500,2000,10^{4}\right\}$, 10000 sweeps + 1000 burn-in.

**Figure 5:**
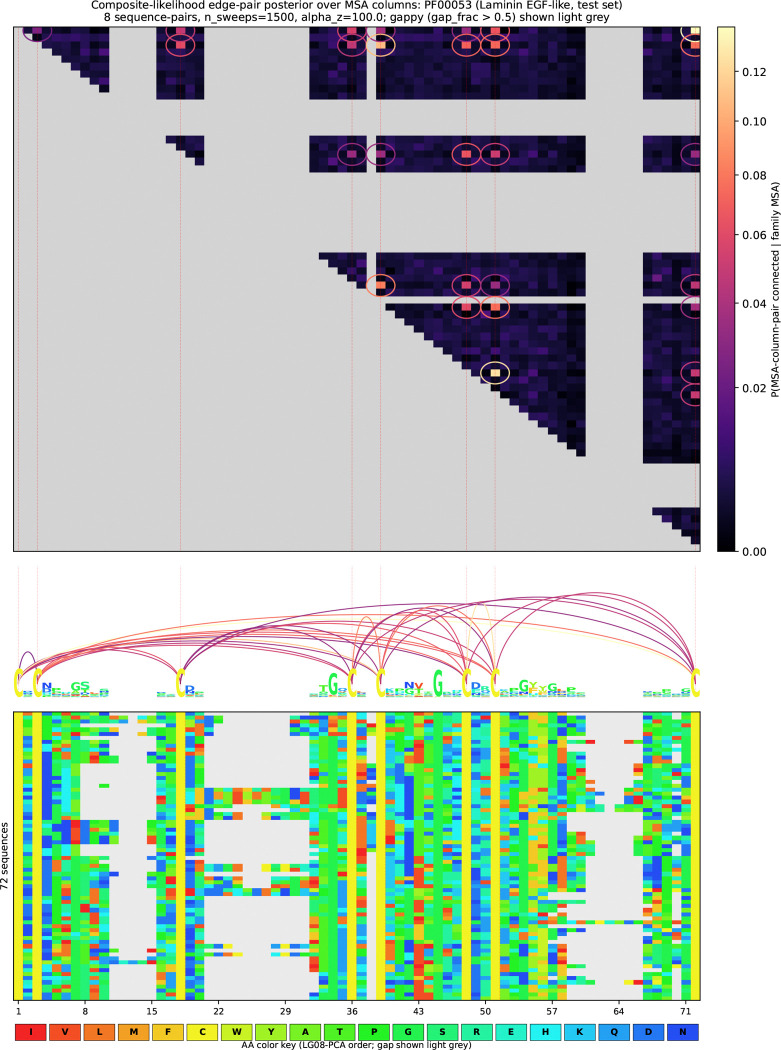
Composite-likelihood MSA-column edge-pair posterior for the held-out PF00053 (Laminin EGF-like) family. Layout from Holmes (11) Fig. 14. *Top:* triangular heatmap *P*(cols ($k_{1},k_{2}$) connected | family MSA) aggregated over 8 sequence pairs by the infinite Pair HMM sampler at the canonical convention. Gappy columns (gap-fraction *>* 0.5) are masked light grey. *Bottom:* the 72-sequence PF00053 MSA coloured by AA chemistry (gap = light grey); red arcs connect the top-scoring column pairs in the triangle above. Cys columns (yellow) at MSA positions {1,3,18,36,39,48,51,72} — the canonical Laminin EGF-like 8-cysteine pattern. The signal is striking: the chain achieves *perfect rank-separation* of Cys–Cys cells from background. With 8 Cys columns there are 82=28 candidate Cys–Cys pairs, and the top-28 cells in the composite posterior are exactly those 28 pairs — every single Cys–Cys pair ranks above every non-Cys cell. Notably, this perfect rank-separation is a property of the *multi-pair composite*; the underlying single-pair posteriors do not achieve it. On the single LAMA1 pair of Figure 4, the minimum-*N* to cover all Cys–Cys cells is *N*=124 for panel (a), *N*=291 for (b), *N*=1402 for (d), and *N*=1500 for (e) — far above the perfect *N*=28 / *N*=45 that the respective C-Cys counts would allow. The composite likelihood averages out per-pair alignment noise; only consistent across-pair Cys–Cys signal survives. Mean Cys–Cys posterior ⟨P⟩=0.060 vs 0.0037 at non-Cys non-gappy background (~16× enrichment) — identical ratio to the single-pair LAMA1 figure (Figure 4). The chain distributes mass across all 28 candidate Cys–Cys pairs without discriminating the 4 *true* disulfide bonds from the other 24. This is the model’s expected behaviour: the per-pair coupling boost $M_{\text{solo}\;}[a,c]$ is position-agnostic (it depends only on the AA pair (a,c)=(C,C), not on the columns), so additional discrimination would have to come from phylogenetic conservation patterns the present composite-likelihood does not yet exploit. Sampler: 1500 sweeps + 300 burn-in per pair, 5-rung RE ladder $\alpha_{z}\in \left\{100,250,700,2000,10^{4}\right\}$.

We ran the canonical infinite Pair HMM sampler on a single sequence pair from human **LAMA1** (laminin subunit α−1, UniProt P25391), a member of the Pfam Laminin EGF-like family PF00053 (*held out from training*): two EGF-like repeats of LAMA1, residues 1452–1506 and 1090–1147, with $L_{x}=55,L_{y}=58,\tau =2.15$ and only 21% sequence identity — diverged enough that the TKF92 baseline alignment is no longer near-deterministic. The joint sampler concentrates posterior mass at Cys–Cys cells at ~17× the background rate on the X axis and ~12× on Y, against ~5× for the single-sequence baselines (Figure 4). The per-pair coupling bonus is weak but detectable; pooled across the 72-sequence family MSA, the composite-likelihood posterior *perfectly* rank-separates all 28 Cys–Cys cell-pairs from the non-Cys background (Figure 5).

## 4 Discussion

TKF-DP introduces exchangeable *nonlocal* coupling structure — DP-clustered Potts interactions between residues that may sit anywhere on the sequence — on top of a single TKF92 indel backbone. The companion MixDom construction (22) and its recursive cousins introduce a different, *local* kind of structure, positional and sequence-ordered, with composition and indel rate varying across a small mixture of latent domains. A realistic generative model of protein evolution should have both, with couplings restricted to within a domain or to specific local contexts: buried-core residues in a globular protein, base-paired residues in an RNA stem, or residues sharing a secondary-structure element. Combining nonlocally- and locally-structured models is a larger technical challenge but well within reach: the recursive grammars of Appendix C provide the natural scaffolding for within-domain or within-element coupling structure.

We made several approximations in our first evaluation of the TKF-DP. Two are worth calling out in detail. (1) We truncated the Ewens prior of Equation (5) to size-{1,2} components; larger Potts components admit an independent-coupling lower bound (N=1 in the midpoint refinement of Appendix D) that extends to a formal ELBO by mesh-refining the midpoint construction, recovering the Cohn fixed point as midpoint spacing tends to zero. (2) We used the standard simplification of treating substitution as conditional on the alignment-visible fragments alone; a more careful treatment would account for the substitution effect on alive sites of transient ghost / gravestone lineages during their lifetimes. We conjecture that this effect can be absorbed into a mean-field correction using the expected trajectory sufficient statistics of Section 2.2, with a heavier importance-sampling treatment available via MCMC from the trajectory SCFG itself.

The hierarchical-DP schema we present here — one DP each for site classes, Potts-atom indices, and key classes — is one design among many; alternative atom-sharing structures, hierarchical class groupings, or supervised training schemes remain unexplored. Further, our use of the DP to induce coupling is transferable to other indel models. For example, the Poisson Indel Process (2) yields a column-independent PIP-DP variant while preserving the DP latent structure.

## Supplementary Material

Supplement 1

Supplement 2 Appendix: roadmap
The technical body of this work — BDI sufficient statistics, exact closed-form Baum–Welch for TKF91/TKF92/MixDom, Maraschino cherry-distillation, the time-indexed gravestone-augmented pair SCFG, the TKF-DP generative graph and inference loop, the infinite Pair HMM MCMC kernel, and the bounded-edge alignment postprocessing family — is collected into five appendices whose contents are largely quoted verbatim from the two companion manuscripts. The roadmap is:
**Appendix A.** BDI and TKF foundations. The finite-state CTMC and linear birth–death–immigration process, the closed-form bridge-expectation and Fisher-score sufficient statistics for TKF91 and TKF92, the singlet-division WFST derivation, the L’Hôpital limits at λ=μ, and a discussion of how TKF92 relates to the latent-boundary-free General Geometric Indel (GGI) model.
**Appendix B.** EM, composite likelihoods, and variational inference. Closed-form substitution M-steps for the many GTR specialisations; the stochastic-variational Baum–Welch loop for TKF91/TKF92 with its ELBO derivation, natural-gradient updates, and convergence theorem; Maraschino, the TKF92 cherry-distilled generalisation of CherryML, and the tree-level inference algorithms it composes with (FSA, BeamASR, VarAnc, svi-VarAnc); and a structural-bias analysis of the BP cumulant under a column-factorised variational field.
**Appendix C.** Recursive TKF. The hierarchical-mixture-of-domains generalisation of TKF92 (MixDom), its exact closed-form M-step via six-step chain-restoration through the fully exploded null-state model, the order-1 Maraschino distillation, the algebraic-distillation family, the SVI-BW convergence considerations specific to MixDom, the tree-level VEM and ancestral-reconstruction algorithms, the generalised phylo-HMM, the labeled-MixDom Singlet and WFST, and the recursive-grammar-elaboration rules that lift these constructions to arbitrary nested-TKF models.
**Appendix D.** TKF-DP: the Dirichlet-process Potts coupling. The generative model of Definition 1, the three-DP latent structure, the class-level path-measure factorisation and constant-rate variational ELBO via pairwise bridge expectations, the augmented indel histories via the time-indexed pair SCFG, the SVI loop, and the pairwise alignment postprocessing landscape (joint-marginal target, coupled annealer, augmented-Pair-HMM family).
**Appendix E.** The infinite Pair HMM and its block-resample MCMC sampler. The three-factor factorisation of Equation (5); the setup-phase $O\left(L^{4}\right)$ partial-Forward precompute; the persweep O(L) block-resample kernel; and the projected lift to the full gravestone-augmented phylogenetic SCFG.

## Figures and Tables

**Table 1:** Alignment accuracy on the 22-family BAliBase BAli3pdbm L < 150 subset (187 pairs).

	$E_{\text{pairs}}\left(Q^{\prime }\right)$		$E_{\text{pairs}}$		$E_{\text{pairs}}$	MSA score	
Method	*F*_1_	*e*_TP_	*F*_1_	*e*_TP_	in MSA *F*_1_	SP	TC

TKF92	0.450	4839	0.475	5202	0.514	0.724	0.631
MixDom-d3f1	0.462	4946	0.489	5372	0.513	0.726	0.638
TKF92-*K*=20	0.458	4921	0.486	5324	0.515	0.732	0.637
CherryML-*C*=20	0.457	4893	0.482	5270	0.515	0.723	0.629
inf-PHMM-*K*=4 (RE)*	0.450	4908	0.475	5232	0.509	0.705	0.609

MAFFT (FFT-NS-2)	—	—	—	—	0.509	0.691	0.558
MUSCLE (default)	—	—	—	—	0.512	0.760	0.662

The L<150 ceiling reflects the practical memory headroom for the infinite-Pair-HMM $O\left(L^{4}\right)$ sampler on an 11 GiB GPU (max-seq-len < 150; see Section 3.2). $E_{\text{pairs}}(Q^{\prime })$ columns are the raw posterior pool; $E_{\text{pairs}}$(opt-acc) is decision-theoretic optimal-accuracy DP applied to the same posterior (13); $E_{\text{pairs}}$ in MSA is the cell-indicator pool from the downstream FSA-merged MSA.

* The infinite-PHMM-*K*=4 row uses the released K4-emwarm-top1000-2026-05-09 TKF-DP checkpoint ($K_{c}=4$ classes, $K_{H}=10$ Potts atoms), with $\alpha_{z}=100$ and corpus-fitted indel rates (λ=0.04581, μ=0.04680,r=0.6835), under replica-exchange (6-rung $\alpha_{z}$ ladder $\left\{100,\;178,\;316,\;562,10^{3},5\times 10^{3}\right\}$, swap every 10 sweeps; 8000 sweeps + 2000 burn-in per rung; 2 RE replicates per pair).

**Table 2:** Alignment accuracy on the full BAliBase bali3pdbm corpus (120 families, all sequence lengths).

	$E_{\text{pairs}}\left(Q^{\prime }\right)$		$E_{\text{pairs}}$		$E_{\text{pairs}}$	MSA score	
Method	*F*_1_	*e*_TP_	*F*_1_	*e*_TP_	in MSA *F*_1_	SP	TC

TKF92	0.289	77,284	0.309	84,913	0.377	0.776	0.622
MixDom-d3f1	0.316	81,324	0.330	89,284	0.384	0.807	0.662
TKF92-*K*=20	0.292	78,140	0.313	85,940	0.378	0.784	0.633
CherryML-*C*=20	0.297	78,633	0.315	86,220	0.384	0.791	0.645

MAFFT (FFT-NS-2)	—	—	—	—	0.365	0.797	0.653
MUSCLE (default)	—	—	—	—	0.373	0.841	0.715

Same methods and column definitions as Table 1 (the L<150 subset), except the infinite-Pair-HMM-*K*=4 row is omitted due to GPU memory constraints on the $O\left(L^{4}\right)$ partial-Forward precompute.
